# The Relationship between a New Biomarker of Vagal Neuroimmunomodulation and Survival in Two Fatal Cancers

**DOI:** 10.1155/2018/4874193

**Published:** 2018-05-08

**Authors:** Y. Gidron, M. De Couck, D. Schallier, J. De Greve, J. L. Van Laethem, R. Maréchal

**Affiliations:** ^1^Vrije Universiteit Brussel, Center for Neuroscience, Brussels, Belgium; ^2^Scalab, Université Lille 3, Lille, France; ^3^Faculty of Health Care, University College Odisee, Aalst, Belgium; ^4^Mental Health and Wellbeing Research Group, Vrije Universiteit Brussel, Ixelles, Belgium; ^5^Oncological Center, UZ Brussels, Jette, Belgium; ^6^Department of Gastroenterology, Erasme University Hospital, Brussels, Belgium

## Abstract

**Background:**

The vagus nerve may slow tumor progression because it inhibits inflammation. This study examined the relationship between a new vagal neuroimmunomodulation (NIM) index and survival in fatal cancers.

**Method:**

We retroactively derived markers of vagal nerve activity indexed by heart rate variability (HRV), specifically the root mean square of successive differences (RMSSD), from patients' electrocardiograms near diagnosis. The NIM index was the ratio of RMSSD to C-reactive protein levels (RMSSD/CRP). Sample 1 included 202 Belgian patients with advanced pancreatic cancer (PC), while sample 2 included 71 Belgian patients with non-small cell lung cancer (NSCLC). In both samples, we examined the overall survival, while in sample 2, we additionally examined the survival time in deceased patients.

**Results:**

In PC patients, in a multivariate Cox regression controlling for confounders, the NIM index had a protective relative risk (RR) of 0.68 and 95% confidence interval (95% CI) of 0.51–0.92. In NSCLC patients, the NIM index also had a protective RR of 0.53 and 95% CI of 0.32–0.88. Finally, in NSCLC, patients with a higher NIM index survived more days (475.2) than those with lower NIM (285.1) (*p* < 0.05).

**Conclusions:**

The NIM index, reflecting vagal modulation of inflammation, may be a new independent prognostic biomarker in fatal cancers.

## 1. Introduction

Despite progress made in treating various cancers, the ability to improve the prognosis in some cancers such as pancreatic cancer (PC) or non-small cell lung cancer (NSCLC) remains grim [1]. For example, the one-year survival rate in stages IIIB–IV lung cancer is poor (30–36%; [2]). Such a situation calls for searching new and modifiable prognostic biomarkers which are also related to key processes affecting prognosis, making such prognostic biomarkers potential therapeutic targets. Recent research suggests that the autonomic nervous system, and particularly the vagus nerve, may affect tumorigenesis. While oxidative stress [3], excessive inflammation [4–6], and sympathetic overactivity [7] all contribute to tumorigenesis, the vagus nerve in contrast inhibits oxidative stress [8], inflammation [9], and sympathetic activity [10]. Due to inhibiting these three tumorigenic factors, the vagus nerve is thought to possibly slow tumor growth [11, 12]. Indeed, several experimental studies in mice, though not all, show that vagotomised tumor-bearing animals had more metastases [13, 14]. Furthermore, one study demonstrated that an anti-inflammatory drug, which depends on the vagus nerve, reduced metastases in tumor-bearing mice [15]. These studies clearly indicate the possible causal relationship between adequate vagal nerve activity and reduced tumor progression, though not all studies point at this direction [16].

In humans, heart rate variability (HRV) is a noninvasive marker of vagal nerve activity [17]. HRV reflects fluctuations in normal R-R interbeat intervals. High HRV predicts lower levels of tumor makers over time [18, 19] and longer survival times in various cancers (e.g., [19, 20]), in most cases independent of known prognostic factors. However, HRV may not predict all outcomes (e.g., presence of metastases), possibly because HRV alone does not consider vagal modulation of inflammation directly. Vagal nerve modulation of inflammation may be the critical link between vagal activity and possible slowing down of carcinogenesis [11]. Indeed, a recent study found that levels of the general inflammatory marker C-reactive protein (CRP) statistically mediated the relationship between HRV and survival time in patients with PC [21]. But can there be one single index which considers both vagal nerve activity and its modulation of inflammation, and does such an index have a prognostic value in cancer? To the best of our knowledge, no study has developed and examined the prognostic value of an index which reflects vagal modulation of inflammation.

Given the possible role of the vagus nerve in modulating cancer via inhibiting inflammation [21], we developed a simple numerical ratio indexing vagal neuroimmunomodulation (NIM) of inflammation. The NIM index considers in one number two systems: vagal nerve activity (the autonomic nervous system in the numerator: HRV) and an inflammatory marker (the immune system response in the denominator: CRP). This study examined in two fatal cancers whether the NIM index (HRV/CRP) could predict survival, independent of confounders. Based on the literature mentioned above, we hypothesized that higher NIM predicts better prognosis, independent of confounders.

## 2. Materials and Methods

### 2.1. Patients

After the approval of the Medical Ethics Committee, medical records of 620 patients with histologically proven advanced (locally advanced and metastatic) pancreatic ductal adenocarcinoma (PDAC) treated at the University Hospital Erasme, Brussels, between 1998 and 2011, and medical records of 650 patients with NSCLC treated at the UZ Brussels hospital between January 2005 and December 2009 were reviewed. Exclusion criteria included conditions known to alter HRV or influence inflammation, such as inflammatory diseases (e.g., arthritis), cardiovascular disease (for the NCSLC patients only), implanted pacemaker, or prescribed cardiologic medication (*β*-blockers, antiarrhythmics for the NSCLC patients only). Following these exclusion criteria and the (un)availability of ECG, CRP, and survival data, 202 PC and 71 NSCLC patients were included.  Table 1 depicts the characteristics of these samples.

### 2.2. Design

The study included a historical prospective design. We collected existing archival electronic patient records (historical) and examined the prospective relationship between the NIM index (see below) derived from these records and measured at baseline (time of diagnosis, time 1), and prognosis at time 2 (e.g., overall survival and survival time). This design is commonly used in the reanalysis of existing data sets.

### 2.3. Measures

#### 2.3.1. Background Variables and Confounders

In both samples, we considered the prognostic roles of patients' age, gender, stage (only in NSCLC), and treatments (chemotherapy, radiotherapy, surgery), as well as whether patients had cardiac problems (only in PC). In PC patients, stage was not considered since all patients had advanced cancer, but we considered whether the tumor spread was locally advanced versus metastatic disease.

#### 2.3.2. Neuroimmunomodulation Index

Heart rate variability (HRV) is a common noninvasive index of vagal nerve activity [17]. We derived the measure of HRV retroactively from 10-second electrocardiographs present in patients' files. Similar ultrashort HRV measures were found to predict tumor markers in previous studies [18, 19] and overall survival in the general population [22]. To index neuroimmunomodulation (NIM), we divided the HRV index of the root mean square of successive RR intervals (RMSSD) by patients' C-reactive protein (CRP), a general marker of inflammation, to yield our NIM index = RMSSD/CRP. Both HRV and CRP were obtained near diagnosis, in both patient cohorts.

#### 2.3.3. Outcomes

In both the PC and NSCLC cohorts, the outcome was the overall survival, defined as the time from the date of histologically proven cancer diagnosis to death or till the end of follow-up for surviving patients. However, in the NSCLC cohort, an additional outcome was the survival time among deceased patients because fewer patients survived. These data were obtained from patients' medical files and the Belgian national registry.

### 2.4. Statistical Analysis

In both cancer samples, the main analysis was a Cox regression survival analysis. After identifying the significant confounders univariately, we entered them together with the categorical NIM index, split at its median value. Time till death or till the end of follow-up (for surviving patients) was also considered as the time variable, and the outcome variable was the vital status. Additionally, in the NSCLC sample, because few patients survived to follow up, we further examined only in the deceased patients the partial correlation between continuous levels of the NIM index and the survival time, controlling statistically for confounders that were significantly related to the survival time.

## 3. Results

**Figure 1:**
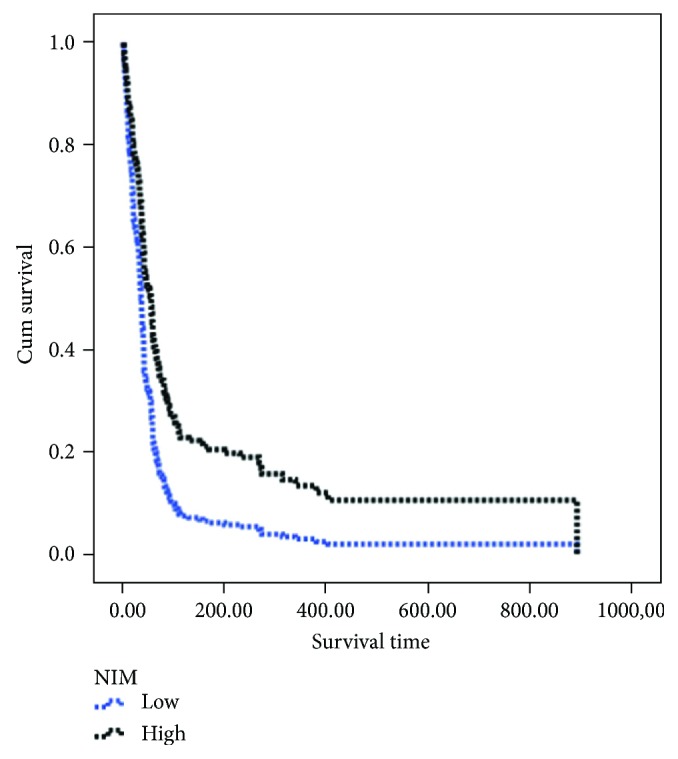
Cumulative survival curves of patients with pancreatic cancer with a high versus low neuroimmunomodulation (NIM) index.

Table 1 presents the background characteristics of each patient cohort. In the PC cohort, 56.9% had locally advanced disease while 43.1% had metastatic tumors. Among all confounders tested, surgery (*p* < 0.001), local versus metastatic disease (*p* < 0.001), cardiac problem (*p* < 0.025), age at diagnosis (*p* < 0.001), and presence of metastatic diseases (*p* < 0.001) were predictive of the overall survival, while radiotherapy and chemotherapy were not (both *p* values > 0.05). Categorical NIM was highly significantly predictive of the overall survival (*p* < 0.001; see Figure 1). In the final multivariate Cox regression (see Table 2), categorical NIM was still significantly predictive of overall survival (*p* = 0.011), independent of the significant confounders mentioned above (for NIM, the relative risk (RR) and 95% confidence interval (CI) were 0.68 and 0.51–0.92, resp.).

**Figure 2:**
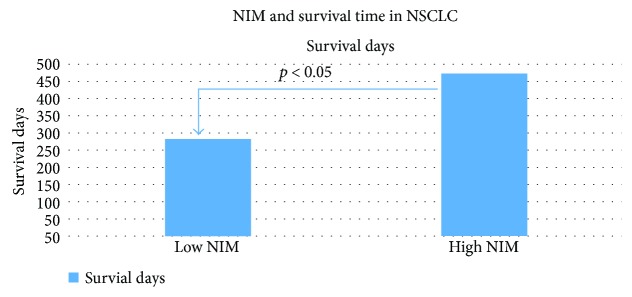
Survival days in deceased patients with non-small cell lung cancer with a high versus low neuroimmunomodulation (NIM) index.

In the NSCLC cohort, among all tested confounders, chemotherapy tended to predict the overall survival (*p* < 0.055), while surgery (*p* < 0.001) and cancer stage (*p* < 0.001) significantly predicted the overall survival. Categorical NIM also significantly predicted the overall survival (*p* < 0.005; see Figure 2). In a multivariate Cox regression (see Table 3), categorical NIM remained a significant predictor of overall survival (for NIM, RR = 0.53; 95% CI: 0.32–0.88). Since many patients in the NSCLC cohort died, we tested the correlation between continuous NIM levels and survival time, in the deceased patients only. Controlling for the above confounders, log-transformed NIM index scores were significantly positively correlated with the survival time (*r* = 0.31, *p* = 0.010). Among these deceased patients, those with a relatively high NIM index (above the median) lived significantly longer (mean (SD) = 475.2 (383.9) days) than those with a low NIM index (mean = 285.1 (282.2) days; *t* (69) = 2.40, *p* < 0.05).

## 4. Discussion

To the best of our knowledge, this may be the first study to develop and test the prognostic value of a new composite biomarker which reflects the autonomic and immune (inflammatory) systems together, in relation to cancer prognosis. Two studies revealed that a new neuroimmunomodulation (NIM) index, which reflects vagal nerve activity (RMSSD) over general systemic inflammation (CRP) significantly predicted the overall survival in patients with advanced PC and in patients with NSCLC. These relationships remained significant also after statistically controlling for confounders which were univariately predictive of survival and which reflected the severity of cancer and its treatment. Patients with NSCLC and a relatively high NIM index had a nearly double survival time compared to those with low NIM.

These results are in line with one experimental study showing that an anti-inflammatory drug (CNI-1493), which depends on and activates the vagus nerve, led to reduced metastases in tumor-bearing mice [15]. These results also support many correlational studies showing that high vagal nerve activity, indexed by HRV, predicts reduced tumor marker levels over time or longer survival, in different cancers including young NSCLC patients (e.g., [18, 19]). These results also support those showing that CRP statistically mediated the relationship between HRV and survival in PC [21]. However, the results of the present study extend these past observations to incorporate the *balance* between vagal activity (RMSSD) and inflammation (CRP) in one ratio and reveal its prognostic value. The novelty in these results is that this new NIM index reveals that a shift in balance from inflammation to vagal activity (a higher NIM ratio) predicts longer survival in two very fatal cancers, independent of relevant prognostic factors. These results are in line with our model of vagal nerve neuroimmunomodulation of cancer [11, 12]. Furthermore, these results are in line with the known anti-inflammatory functions of this important cranial nerve [9]. This index includes HRV derived from ECGs and includes CRP levels, both which are routinely available worldwide in hospitals. This availability makes the new NIM index highly feasible for use to estimate cancer patients' prognosis. Finally, using noninvasive vagal nerve activating methods (e.g., electric vagal nerve stimulation; [23]), NIM is a marker which could be therapeutically modified, making this index also a possible therapeutic target on its own, to possibly then influence prognosis. Future controlled intervention studies need to test such treatment avenues.

This study had several limitations. First, we did not employ formal prospective studies, rather historical prospective designs. However, previous studies demonstrating prognostic values of HRV used similar designs (e.g., [19]). Furthermore, the EGCs, from which HRV was derived, and CRP were both measured near diagnosis, while follow-ups were done later, maintaining a longitudinal aspect in the design. Second, the ECGs were very brief—lasting only 10 seconds. However, multiple studies including one large population study have demonstrated that such brief HRV measures have a prognostic value [18, 19, 21, 22] and that this brief measure correlates with HRV measures in longer tests [24]. In addition, we showed here the prognostic value of the NIM index in two cancer samples, attesting to the reliability of this new observation. Nevertheless, these results should be replicated in a formal prospective study, with longer ECG readings. Should the results be replicated, and should the new NIM index be found to be predictive in other cancers (and chronic diseases), this NIM index may be proven clinically important for prognostication and for identifying patients who require more close medical care. Finally, the relationship between NIM and prognosis only reflects an association, not a causal relationship between these variables. Some of the animal studies conducted to date do propose a causal relationship between vagal nerve activation and improved cancer prognosis [13–15]. To test this important issue in people, investigators need to test whether activating the vagus nerve shifts the balance from inflammatory to vagal activity and whether this shift could then improve cancer treatment and patients' prognosis, the ultimate goal. These would have both scientific and potential clinical significances.

## Figures and Tables

**Table:** (a) Categorical variables

Variable	PC (%)	NSCLC (%)
Gender		
Men	53.0%	63.4%
Women	47.0%	36.6%
Radiotherapy	13.4%	69.0%
Chemotherapy	73.3%	76.0%
Surgery	40.1%	10.0%
Stage		
Stage 1		11.3%
Stage 2		7.0%
Stage 3		21.1%
Stage 4		60.6%

**Table:** (b) Continuous variables

Variable	PC Mean (SD)	NSCLC Mean (SD)
Age (years)	66.2 (11.8)	62.5 (11.5)
HRV (RMSSD)	27.4 (20.5)	21.6 (24.0)
CRP	4.7 (18.8)	43.1 (63.3)

PC: pancreatic cancer; NCSLC: non-small cell lung cancer; HRV: heart rate variability; RMSSD: root mean square of successive differences; CRP: C-reactive protein.

**Table 2:** Multivariate Cox regression survival analysis of relative risks for death in patients with advanced pancreatic cancer.

Variable	B	Sig.	RR	95% CI
Surgery	−0.84	0.00	0.43	0.30–0.62
Local/advanced	−0.09	0.71	0.91	0.55–1.51
Cardiac problem	0.13	0.41	1.14	0.83–1.57
Age	0.02	0.01	1.02	1.00-1.03
Metastases	0.28	0.30	1.33	0.77–2.28
NIM index	−0.38	0.01	0.68	0.51–0.92

Sig.: significance; RR: relative risk; 95% CI: 95% confidence interval; NIM index: neuroimmunomodulation index.

**Table 3:** Multivariate Cox regression survival analysis of relative risks for death in patients with non-small cell lung cancer.

Variable	B	Sig.	RR	95% CI
Chemotherapy	−0.44	0.25	0.64	0.30–1.38
Surgery	−1.20	0.01	0.30	0.11–0.79
Stage	0.48	0.01	1.61	1.24–2.10
NIM index	−0.63	0.01	0.53	0.32–0.88

Sig.: significance; RR: relative risk; 95% CI: 95% confidence interval; NIM index: neuroimmunomodulation index.
